# How healthy is community-dwelling elderly population? Results from Southern Italy

**Published:** 2016-01-31

**Authors:** Francesca Guerriero, Valentina Orlando, Daniele Ugo Tari, Annalisa Di Giorgio, Antonio Cittadini, Gianluca Trifirò, Enrica Menditto

**Affiliations:** 1 CIRFF, Center of Pharmacoeconomics, Federico II University of Naples, Italy;; 2 Caserta Local Health Unit,Caserta, Italy;; 3 Department of Traslational Medical Science, Federico II University of Naples, Italy;; 4 Department of Clinical and Experimental Medicine, University of Messina, Italy;

**Keywords:** Polypharmacy, elderly, administrative databases, frailty, geriatric assessment

## Abstract

**PURPOSE:**

To explore the frequency of polypharmacy, functional and cognitive capacity among the elderly in Southern Italy.

**METHODS:**

Population-based retrospective cross-sectional study. Information were retrieved from electronic-geriatric-forms matched by record-linkage to outpatient pharmacy-records. The following domains were collected from geriatric forms: BMI, cognitive capacity (SPMSQ), functional status (Barthel-index), mobility, living condition. Polypharmacy status was categorized as non-polypharmacy (0–4), polypharmacy (5–9) and excessive-polypharmacy (≥10). Prevalence of all variables were stratified by age and polypharmacy group.

**RESULTS:**

88,878 old people received a geriatric assessment in the years 2013–2014. Mean age was 74.8 (±7.3) years, 56.6% females. Proportion of elderly in excessive-polypharmacy increased with age (18.9% in 65–75 age-group; 27.9% in >85). Referring to cognitive capacity, the proportion of lucid patients decreased with age (from 94.3% to 58.1%), while confused patient increased with age (from 4.7% to 30.9%). Proportion of subjects with a decline in cognitive status, functional status and mobility increased in polypharmacy and excessive polypharmacy group.

**CONCLUSION:**

Polypharmacy is common in people aged 65 years and older with difficulties in activities of daily living and impaired cognition. Furthermore, its prevalence raises with increasing age. Preventive strategies such us optimization of drug regimen should be performed routinely to reduce risk of adverse-health-events.

## I. INTRODUCTION

Polypharmacy is one of the most relevant health-related issues in elderly population. Drug treatment may influence both positively and negatively elderly health status. Polypharmacy increases the risk of inappropriate prescribing, drug–drug and drug–disease interactions, and hence the risk of adverse health events including falls, functional impairment, and hospitalization [1–2].

Nowadays, different sets of indicators have been developed in order to provide a measure of prescribing performance and, hence, to assess quality of care in older people [3–4]. However, this may not be sufficient for frail elderly who have several problems related to the functional status, mobility, cognitive status and living condition. In these patients, the most appropriate approach to re-evaluate the drug- therapy should combine evidence-based data with information gathered from a multidimensional geriatric assessment. Recently, Multidimensional Assessment Schedule (SVaMA) was developed to effectively explore multiple domains of health, as multidimensional and multidisciplinary tool of choice to determine the prognosis of the functionally compromised and frail older subject [5]. SVaMa includes information on functional (activities of daily living, ADL, and Instrumental ADL), cognitive status (Short Portable Mental Status Questionnaire), nutrition (Mini Nutritional Assessment), comorbidities (Cumulative Illness Rating Scale), medications and co-habitation status (i.e. living alone or with someone).

In 2012 the European Commission launched an initiative called ‘European Innovation Partnership on Active and Healthy Aging’ (EIP-AHA) with the purpose of stimulating research and innovation in EU and increase understanding around frailty and the prevention, early diagnosis and management of functional decline, both physical and cognitive, in older people [6].

Since 2013 Campania Regional Health-Care System introduced electronic geriatric forms available to general practitioners to perform a multidimensional assessment on community-dwelling older patients. The aim of this population-based retrospective study was to explore the frequency of polypharmacy and the functional status of older outpatients from Caserta Local Health Unit (LHU), Southern Italy.

## II. METHODS

**Study design**: we designed a retrospective cross-sectional study.

**Setting:** electronic geriatric forms from the Local Health Authority (LHU) of Caserta in the Campania Region (Southern Italy), covering a population of about 1,000,000 inhabitants.

### Data sources

The data used for this study were obtained from electronic geriatric forms in the SANIARP Portal, a web platform available to general practitioners (GPs) of LHU Caserta, collected from January 1, 2013 to December 31, 2014 (study period). Electronic geriatric card was a short version of SVaMA. The SVaMA is the officially recommended assessment schedule used by the health personnel of the National Health Care System to perform a multidimensional assessment on community-dwelling older persons or nursing home residents to establish accessibility to some health care resources [5]. From the electronic geriatric cards the following information were obtained: Body Mass Index (BMI); cognitive capacity (Short Portable mental Status Questionnaire SPMSQ); functional status (Barthel index); mobility (Barthel index), living condition and senses and communication skills (language understanding, language production, hearing and sight). This data source was matched, by record-linkage analysis to outpatient pharmacy records and the civil registry in order to collect pharmaceutical information (number of drugs) and demographic information (i.e. age, gender, date of death or emigration) of all residents covered by the LHU. All information was linked through a unique and anonymous personal identification code. As this automated system is anonymous, neither ethical committee approval nor informed consent was required. Furthermore, the anonymous data file is routinely used by the local health authority for epidemiological and administrative purposes. Permission to use it for the present study was granted by the responsible authority. The reliability of this strategy to produce pharmacoepidemiological information has been previously documented [7–8].

### Study population

The study population consisted of all subjects of 65 years of age or older who had at least one geriatric assessment form during the study period (January, 1 2013 to December, 31 2014). We considered the latest form for patients with more than one geriatric assessment. Age was calculated at the date of geriatric assessment was carried out.

### Variables

***Short Portable mental Status Questionnaire SPMSQ*** is a short questionnaire that is used to screen older adults for dementia signs and other neurologically based deficits and to determine the degree of impairment [9].

***Functional status*** was evaluate **by Barthel scale or Barthel Index.** This is an ordinal scale used to measure performance in activities of daily living (ADL). Each performance item is rated on this scale with given number of points assigned to each level or ranking [10]. The rating scale autonomy in basic activities of daily living proposal by Katz et al. is one of the tools used in the evaluation of geriatric patient [11]. The tool evaluates accurately basic tasks: to bathe, dressing up, toilet, move, urinary and fecal incontinence, feed themselves. The index measures the different abilities of the patient in taking care of themselves and each is measured in terms of what the patient is functional or not. To each of the items goes given a score dichotomous in which: 0 = dependent 1 = independent

***Mobility*** was assessed by four tasks: Transfer bed / chair, walk, use of wheelchair, use the stairs. Each performance item is rated on the Barthel index. To each of the items goes given a score dichotomous in which: 1 = moves by self’s 2= assisted [12].

***Living condition*** was determined on the basis of living condition at the time of examination. Living status was coded for those participants living alone or with someone. (Alone = 1; with someone = 2) [13].

***Language*** (understanding and production) was categorized into 3 groups: Normal, Understand only simple sentences or disorder of language, doesn’t understand doesn’t speak respectively.

***Hearing and Sight*** was categorized into 4 groups: Normal, Serious deficit, Serious deficit incurable and finally deafness or blindness.

***Polypharmacy*** was defined as a three-class variable: *excessive polypharmacy* defined as the use of ten or more drugs; *polypharmacy* as the use of five to nine drugs; *nonpolypharmacy* as the use of four or less drugs concomitantly [14].

Prevalence of all variables were stratified by age-group (65–75 years; 75–84 years; >85 years) and polypharmacy group (non-polypharmacy; polypharmacy; excessive polypharmacy).

### Statistical methods

Characteristics of the study population were analysed using descriptive statistics: quantitative variables were described by means and standard deviations while categorical variables were described by counts and percentages. In the case of categorical variables, crosstabulations with chi-square tests were used for comparing the differences between age group and polypharmacy group. All analyses were performed using SPSS software version 17.1 for Windows (SPSS Inc., Chicago, IL, USA). Statistically significance was set up at p-value < 0.05

## III. RESULTS

A total of about 90,000 elderly were analysed in this study. This amount represents more than 60% of the total elderly population in LHU Caserta. The mean patient age at the index date was 74.8 (±7.3) years with 53.7% of the patients between 65–74 years of age, 34.6% of the patients between 75–84 years of age, 11.7% of patients 85 of age or more. Baseline characteristics of the study population are shown in Table 1. 56.6% of patients were women. The mean BMI of patients was 27.0 (± 4.0). In particular, percentage of overweight elderly decreased from 43.0% in subjects aged 65–74 years to 35.8% in subjects aged 85 or more. Proportion of elderly in polypharmacy increased from 42.7% in subject aged 65–74 years to 45.4% in subject aged 85 or more. More in detail, with regard to excessive polypharmacy, there was a slightly increase from 18.9% in subject aged 65–74 years to 27.9% in subject aged 85 or more. Referring to cognitive capacity, the proportion of confused patients increased from 4.7% in patients aged 65–74 years to 30.9% in patients aged 85 or more. The percentage of very confused patients increased from 0.9% in patients aged 65–74 years to 11.0% in patients aged 85 or more. In the matter of functional status, the percentage of patients dependent in their activities of daily living rose from 2.9% in patients aged 65–74 years to 36.2% in patients aged 85 or more. Therefore, about mobility, the proportion of elderly needing help to move increased from 5.3% in subject aged 65–74 years to 48.7% in subject aged 85 or more. Overall, 87.5% of elderly living with someone and only 12.5% living alone. Regarding senses and communication skills, the proportion of elderly having problems with language, hearing and sight increased proportionally with the age. In particular, the percentage of elderly who “understand only simple sentences” and “disorder of language” increased from 6.7% and 9.0% in subject aged 75–84 years to 17.6% and 22.4% in subject aged 85 or more, respectively. Therefore, referring to hearing and sight the percentage of elderly with serious deficit increased from 17.8% and 29.2% in subject aged 65–74 years to 58.3 and 62.2% in subject aged 85 or more, respectively.

Approximately 67.4% of elderly are treated with five or more drugs. In particular, the proportion of elderly patients in polypharmacy, defined as the use of five to nine drugs, was 44.2% and 23.2% was in excessive polypharmacy, defined as the use of more than ten drugs, as shown in Table 2. Proportion of subjects with a decline in cognitive status, functional status and mobility increased in polypharmacy and excessive polypharmacy group. Referring to SPMSQ score, the percentage of confused patients increased from 8.0% in non-polypharmacy group to 15.6% in excessive polypharmacy group. The percentage of very confused patient rose from 2.3% in non-polypharmacy group to 4.4% in excessive polypharmacy group. The same trend in proportion occurred for functional status and mobility. In particular, the proportion of elderly dependents increased in all three groups of polytherapy (5.4%; 9.1%; 17.0% respectively). The percentage of elderly assisted increased from 8.3% in non-polypharmacy group to 25.6% in excessive polypharmacy group.

## IV. DISCUSSION

This population-based study investigated the frailty status, assessed through multidimensional evaluation of different domains, in a large population of elderly outpatients by using electronic geriatric forms in the SANIARP portal, a web platform available to GPs of LHU Caserta.

Overall, the study showed that polypharmacy status was very frequent in our population, in detail about 44% of elderly patients received between five and nine drugs and 23.2% took more than ten drugs. While the data regarding polypharmacy (5–9 drugs) are in line with findings from Italian National Agency (AIFA) [15], we found a higher percentage of elderly in excessive polypharmacy 23% vs 11%. This could be due to the different data sources as we analysed not only drugs dispensed by community pharmacies but also drugs dispensed directly by hospital pharmacies. Furthermore underlying population and observation period of the analysis was different. Our findings are clinically relevant insofar as polypharmacy is associated with a higher risk of poor health outcomes such as falls, avoidable hospitalization [16–18]. Furthermore the excessive polypharmacy is associated with decline in nutritional status, functional ability and cognitive capacity in elderly persons aged 75 years and older [19]. Apart from polytherapy we also analysed decline in cognitive status, functional status and mobility. Our findings showed that subjects aged 85 or more 30.9% were confused, 36.2% were dependent, 48.7 % were assisted and about 20% had a disorder of language. All these conditions may affect aspects related to therapeutic appropriateness followed by the patient: our findings showed that about 28% of highest age category received more than nine drugs. A recent study conducted by Herr et al. estimated that each additional drug prescribed was associated with an increased risk of being pre-fail or dependent, with adjusted OR 1.12 (95% CI 1.07–1.17), 1.20 (95% CI 1.12–1.128) and 1.26 (95% CI 1.17–1.35), respectively [20].

The study evaluated only polypharmacy status but not wise use of drug therapy so we cannot state whether the 27% of oldest old in polypharmacy is treated appropriately or not. However it must be kept into account that the coexistence of clinical complexity along with the lack of evidence on drug effectiveness from clinical trials in very old persons, does not provide physicians with knowledge on outcomes associated with an aggressive pharmacological treatment.

In view of this evidence, preventive strategies should be devised for old people with regard to physical activity, cognitive status and management of chronic diseases. The optimization of polypharmacy is part of these actions. Different approaches to reduce unnecessary medication use in elderly have been considered, involving pharmacists or geriatricians, using implicit or explicit criteria. Nevertheless, research is still needed to determine the most efficient strategies [21–22].

It is important to outline that there is a lack of consideration of the frail status in therapeutic guidelines although some age-related conditions may have an impact of late-life influencing, such as frailty [23].

Frailty in older people reflects a nonspecific state of vulnerability and a multisystem physiological change with increased risk for adverse health outcomes in older age [24]. In fact, frailty is acknowledged to be a multidimensional concept associated with a greater risk for adverse health-related outcomes such as falls, disability, hospitalization, permanence institutionalization and death [25]. Our study was limited to describe several domains that can influence frailty status. We did not define a score to assess this aspect. In fact available information are only a part of the complete SvaMa. However we described some of the conditions that should be considered in the evaluation and revision of therapy in elderly patients.

It would be interesting to define frailty scores from our data in order to achieve a better use of patient information. The most recent evidences highlighted how it is possible to calculate, on the basis of validated algorithms, from SVAMA the Multidimensional Prognostic Index (MPI) able to predict the risk of mortality. The clear advantage of this instrument can result in a significant chance to reduce mortality, functional disability and cognitive impairment of older subjects, according to the most recent evidence in clinical geriatric practice [26–27].

The strength of our study is that it was possible to match data coming from different sources (administrative database and electronic geriatric cards in the SANIARP Portal) at single patient level. In this way, we were able to collect information about the functional status, cognitive status and mobility according to drug prescription records, while keeping into account relevant personal details such as sex and age. On the other hand, information about the type of drugs taken by elderly patients and the presence of comorbidities were not investigated as the aim of this study was evaluate level of polypharmacy and excessive polypharmacy according to other domains relevant in the evaluation of drug therapy in the elderly such as functional, cognitive and mobility status. The present initiative is part of the strategy carried out by the EIPAHA A1 Action Group on adherence and provides preliminary data that might be useful for more focused interventions. Moreover it can represent a point of synergy with EIP-AHA A3 Action Group on frailty as polypharmacy has also great relevance in assessment of vulnerability of elderly patients.

## V. CONCLUSION

Our study highlighted that polypharmacy is more frequent in older patients with a decline in cognitive status, functional status and mobility. All these conditions, associated with polypharmacy regimen can influence frailty status and can affect treatment outcomes with a greater risk for adverse health-related outcomes. Our findings emphasize how important are preventive strategies such as optimization of drug regimen for a better and safer management of elderly patients.

## Figures and Tables

**Table 1.** **Baseline characteristics of the study population**

**Variables**	**Age groups, N (%)**			**Total, N (%)**	

	**65–74 years** 47,741 (53.7)	**75–84 years** 30,777 (34.6)	**≥85 years** 10,360 (11.7)	88,878 (100.0)	***p***
**Gender**					*<0.001*
Female	25,292 (53.0)	17,962 (58.4)	7,057 (68.1)	50,311 (56.6)	
Male	22,449 (47.0)	12,815 (41.6)	3,303 (31.9)	38,567 (43.4)	
**BMI**					*<0.001*
Mean (±SD)				27.0 (±4.0)	
Underweight (BMI≤18.49)	66 (0.1)	59 (0.2)	43 (0.4)	168 (0.2)	
Normal (BMI ≥18.50 ≤24.99)	15,385 (32.2)	9,998 (32.5)	4,097 (39.5)	29,480 (33.2)	
Overweight (BMI ≥25)	20,529 (43.0)	12,655 (41.1)	3,714 (35.8)	36,898 (41.5)	
Obese (BMI ≥30)	8,610 (18.0)	5,957 (19.4)	1,873 (18.1)	16,440 (18.5)	
**Number of drugs**					*<0.001*
Non-polypharmacy (0–4 drugs)	18,308 (38.3)	7,903 (25.7)	2,764 (26.7)	28,975 (32.6)	
Polypharmacy (5–9 drugs)	20,405 (42.7)	14,166 (46.0)	4,702 (45.4)	39,273 (44.2)	
Excessive Polypharmacy (≥10 drugs)	9,028 (18.9)	8,708 (28.3)	2,894 (27.9)	20,630 (23.2)	
*SPMSQ score					*<0.001*
Lucid (0–3)	45,041 (94.3)	25,213 (81.9)	6,022 (58.1)	76,276 (85.8)	
Confused (4–8)	2,252 (4.7)	4,464 (14.5)	3,199 (30.9)	9,915 (11.2)	
Very confused (9–10)	448 (0.9)	1,100 (3.6)	1,139 (11.0)	2,687 (3.0)	
**Functional status (Barthel Index)**					*<0.001*
Indipendent (0–14)	46,373 (97.1)	27,271 (88.6)	6,609 (63.8)	80,253 (90.3)	
Dipendent (15–49)	1,368 (2.9)	3,506 (11.4)	3,751 (36.2)	8,625 (9.7)	
**Mobility (Barthel Index)**					*<0.001*
Moves by itself (0–14)	45,225 (94.7)	25,016 (81.3)	5,318 (51.3)	75,559 (85.0)	
Assististed (15–49)	2,516 (5.3)	5,761 (18.7)	5,042 (48.7)	13,319 (15.0)	
**Living_condition**					*<0.001*
Live alone	4,905 (10.3)	4,857 (15.8)	1,350 (13.0)	11,112 (12.5)	
Live with someone	42,836 (89.7)	25,920 (84.2)	9,010 (87.0)	77,766 (87.5)	
**Language (Understanding)**					*<0.001*
Normal	46,477 (97.4)	28,120 (91.4)	7,946 (76.7)	82,543 (92.9)	
Understand only simple sentences	1,000 (2.1)	2,055 (6.7)	1,819 (17.6)	4,874 (5.5)	
Doesn’t understand	225 (0.5)	815 (2.6)	595 (5.7)	1.461 (1.6)	
Normal	46,114 (96.6)	27,721 (90.1)	7,774 (75.0)	81,609 (91.8)	
Disorder of language	1,479 (3.1)	2,759 (9.0)	2,319 (22.4)	6,557 (7.4)	
Doesn’t speak	148 (0.3)	297 (1.0)	267 (2.6)	712 (0.8)	
**Hearing**					*<0.001*
Normal	38,731 (81.1)	18,528 (60.2)	3,443 (33.2)	60,702 (68.3)	
Serious deficit	8,513 (17.8)	11,338 (36.8)	6,042 (58.3)	25,893 (29.1)	
Serious deficit incurable	360 (0.8)	726 (2.4)	702 (6.8)	1,788 (2.0)	
Deafness	137 (0.3)	185 (0.6)	173 (1.7)	495 (0.6)	
**Sight**					*<0.001*
Normal	33,344 (69.8)	16,357 (53.1)	3,352 (32.4)	53,053 (59.7)	
Serious deficit	13,931 (29.2)	13,735 (44.6)	6,445 (62.2)	34,111 (38.4)	
Serious deficit incurable	384 (0.8)	583 (1.9)	475 (4.6)	1,442 (1.6)	
Blindness	82 (0.2)	102 (0.3)	88 (0.8)	272 (0.3)	

* SPMSQ : Short Portable Mental Status Questionnaire

**Table 2.** **Mobility, functional status and cognitive status (N, %) stratified by polypharmacy group.**

**Variables**	**Polypharmacy group**, N (%)			**Total**, N (%)	

	**0–4 drugs** 28,975 (32.6)	**5–9 drugs** 39,273 (44.2)	**>10 drugs** 20,630 (23.2)	88,878 (100.0)	***p***
SPMSQ* score					<0.001
Lucid (0–3)	26,003 (89.7)	33,769 (86.0)	16,504 (80.0)	76,276 (85.8)	
Confused (4–8)	2,317 (8.0)	4,376 (11.1)	3,222 (15.6)	9,915 (11.2)	
Very confused (9–10)	655,0 (2.3)	1,128 (2.9)	904,0 (4.4)	2,687 (3.0)	
**Functional status (Barthel Index)**					<0.001
Indipendent (0–14)	27,422 (94.6)	35,705 (90.9)	17,126 (83.0)	80,253 (90.3)	
Dipendent (15–49)	1,553 (5.4)	3,568 (9.1)	3,504 (17.0)	8,625 (9.7)	
**Mobility (Barthel Index)**					<0.001
Moves by itself (0–14)	26,560 (91.7)	33,649 (85.7)	15,350 (74.4)	75,559 (85.0)	
Assisted (15–49)	2,415 (8.3)	5,624 (14.3)	5,280 (25.6)	13,319 (15.0)	

* SPMSQ : Short Portable Mental Status Questionnaire
